# Impact of income inequality on breast cancer mortality according to socioeconomic status in the Federative Units of Brazil

**DOI:** 10.3389/fpubh.2022.972204

**Published:** 2022-09-28

**Authors:** Katia Pereira Tomaz, Samantha Hasegawa Farias, Wilson Leite Maia Neto, Francisco Winter dos Santos Figueiredo, Fernando Adami

**Affiliations:** ^1^Epidemiology and Data Analysis Laboratory, Centro Universitário Faculdade de Medicina do ABC (FMABC), São Paulo, Brazil; ^2^Faculty of Collective Health, Federal University of the South and Southeast of Pará Unifesspa, Marabá, Brazil; ^3^Department of Medicine, Instituto Tocantinense Presidente Antonio Carlos - ITPAC, Palmas, Brazil

**Keywords:** breast cancer, mortality, income inequality, socioeconomic status, epidemiology

## Introduction

Cancer is a group of more than 100 diseases that have in common the disordered growth of cells that invade tissues and organs, and can spread to other regions of the body. With quickly multiplication, these cells tends to be very aggressive and uncontrollable, determining the formation of tumors or malignant neoplasms. The causes of cancer can be external and internal to the organism, being interrelated (1).

Breast cancer is considered a public health problem, being the first worldwide cause of mortality in women (2). Tiezzi (3) reports in his study that developing countries are decreasing the rate of breast cancer due to changes in habits in quality of life. In developed countries, even though there is prevention through screening, the prevalence is still high, the World Health Organization (WHO) estimates that this prevalence can be attributed to overweight and obesity.

By the year 2030, the annual rates of breast cancer in the world will have reached 2.7 million cases and around 870 thousand deaths (4). Administrative areas with better socioeconomic status tend to concentrate higher diagnosis rates. However, the death rates are greater in underdeveloped areas (5). This contrast might change in the future, since the occurrence of this kind of cancer is expected to increase in developing and underdeveloped countries due to lifestyle and behavioral changes (4). In the face of the combination between socioeconomic and epidemiological scenarios, the analysis of the vulnerability indexes, as well as the access to a private or public health care system, can provide a resource for understanding how new occurrences, previous occurrences and deaths caused by this cancer affect different layers of society.

In Brazil, the relationship between socioeconomic development and breast cancer rates among administrative regions appears to follow a similar trend to that of developed countries (6). However, the last decade has shown a reduction in income inequality in Brazil, which represents a different situation from that observed in developed countries and a unique scenario among developing countries (7). The improvements in quality of life, access to health care and better socioeconomic conditions can affect different populations in various ways. The gap between opposite social extracts might shrink, but the impact of such transformations won't necessarily happen in the near future (8).

Therefore, the objective of this study was to relate income inequality and epidemiological indicators of breast cancer in women, according to socioeconomic levels of the Brazilian Federative Units.

## Methods

### Study design

This is an ecological study with data from the federative units of Brazil, conducted in the year 2020 with secondary data referring to 2017, obtained through the Global Burden of Diseases (GBD) information system.

### Source of data

The Global Burden of Diseases (GBD) is an initiative of the Institute for Health Metrics and Evaluation (IHME) group that analyzes the updated estimates of world's health for 359 diseases and injuries, and 84 risk factors from 1990 to 2017, among then breast cancer mortality that we will use in this study for the federative units of Brazil in 2017 (9).

The income inequality indicators used included the Gini index and Human Development Index (HDI), obtained from the social vulnerability atlas (10).

### Study variables

#### Income inequality

The exposure variable taken by this study was income inequality, obtained by the gross value of the Gini Index extracted from the Atlas of Social Vulnerability (IVS). The Gini index is a measure of income inequality that ranges from 0 to 1, where presenting a lower value is related to greater income distribution, and therefore lower income inequality (6).

#### Socioeconomic status

The Municipal Human Development Index (HDI) is a three dimensional measurement indicators of human development: longevity (measured life expectancy at birth), education (composition of schooling indicators for the adult population and the school flow of the young population), and income (municipal income per capita). The index ranges from 0 to 1. The closer to 1, the higher the human development (11).

In this present study, in order to classify federative units according to socioeconomic levels, we analyzed the distribution of MHDI values in tertiles, with the first tertile of distribution being the federative units with the lowest level of development, the second tertile as moderate socioeconomic level and the third tertile as a high socioeconomic level.

#### Epidemiological indicators of breast cancer in women

Epidemiological indicators used were Mortality, Incidence, Prevalence, Disability-adjusted life years (DALYs), Years of healthy life lost due to disability (YLDs) and Years of life lost to due to premature mortality (YLLs). Were extracted from the Global Burden of Diseases (GBD), for the lastest year available in the system during the time of data collection (2017), with the Tenth International Classification of Diseases (ICD-10) by code C50 (12). All breast cancer indicators were extracted for women only, standardized by age using the GBD 2017 World Standard Population and as a rate per 100,000 inhabitants (13).

### Statistical analysis

Descriptive statistics were performed by absolute values. To perform the analytical statistics, initially the distribution of the data was verified using the Shapiro-Wilk test, considering as normal distribution data the results of the respective test with the value of *p* ≥ 0.05.

To quantify the association between breast cancer and the Gini index, linear regression was performed, stratified by socioeconomic levels and adjusted by per capita income values. Adjustment by per capita income for analyzes of income inequality is necessary to decrease the bias of studying the effects of income rather than the effects of income inequality (14).

To analyze the effects of socioeconomic status on the relationship between income inequality and epidemiological indicators of breast cancer in women, two analysis models were developed. The first model evaluates the relationship between the HDI, per capita income and epidemiological indicators. The second model adds the income inequality indicator to the first model to test our hypothesis that the issues related to income inequality can impact the outcomes and alter this relation.

In all models, the slope (β) with the respective confidence interval, the predictive capacity (*r*^2^) and the respective p-values (p-value) were estimated. We have used the Pearson correlation to test the collinearity between the predictive variables of the model. The residual analysis was carried out at the end of the adjustment to verify if the normality and heteroscedasticity assumptions were respected. The established significance level was 5% and the program used for statistical analysis was Stata 11.0^®^ (StataCorp, LC).

### Ethical aspects

According to Resolution No. 510, of April 7, 2016 of the National Health Council of Brazil, research using publicly available information should not be evaluated by the CEP/CONEP system, as is the case with the present research (15).

## Results

It was observed that among the Federative Units classified as low socioeconomic level in 2017, Sergipe had the worst indicators related to breast cancer in women. Additionally, Pernambuco among those classified as moderate socioeconomic level and Rio de Janeiro, classified as high socioeconomic level, were the ones that stood out with the worst indicators of this type of cancer (Table 1).

**Table 1:** Standardized rates of epidemiological indicators of female breast cancer (per 100 thousand inhabitants) in the federative units of Brazil, 2017.

**Socioeconomic levels/Federative units**	**Mortality**	**Incidence**	**Prevalence**	**DALYs**	**YLDs**	**YLLs**
**Low**						
Bahia	12.12	31.75	253.57	378.90	18.70	360.19
Maranhão	9.22	21.69	171.87	273.68	12.68	261.01
Pará	10.31	25.53	202.80	304.74	14.95	289.79
Sergipe	13.56	34.74	272.95	404.14	20.27	383.87
Alagoas	11.60	28.20	218.99	347.90	16.45	331.45
Acre	9.14	21.90	172.98	261.43	12.77	248.65
Piauí	10.26	25.93	207.52	313.61	15.26	298.35
**Moderate**						
Amapá	9.59	24.57	195.54	277.91	14.50	263.41
Roraima	11.57	28.27	216.99	305.37	16.32	289.05
Ceará	13.30	34.25	271.61	396.94	20.05	376.88
Mato Grosso do Sul	13.60	36.79	290.52	398.71	21.64	377.06
Pernambuco	15.31	37.79	291.43	448.36	21.91	426.45
Espírito Santo	12.34	34.96	280.32	368.92	20.72	348.20
Mato Grosso	11.03	29.79	238.02	323.36	17.58	305.79
Tocantins	10.06	26.78	213.99	299.59	15.79	283.80
Rondônia	10.51	28.19	224.00	307.46	16.61	290.85
Rio Grande do Norte	13.26	36.38	289.87	395.85	21.43	374.41
Amazonas	11.60	30.39	240.9	345.43	17.83	327.60
Goiás	11.98	32.96	264.31	257.80	19.50	338.31
Paraíba	12.71	33.00	261.10	375.08	19.33	335.75
**High**						
Rio de Janeiro	19.84	56.72	444.73	579.95	33.60	546.35
Santa Catarina	15.24	47.27	379.88	448.67	28.19	420.48
Paraná	15.41	43.94	347.53	447.12	25.93	421.19
São Paulo	16.06	49.96	402.09	466.40	29.91	436.49
Minas Gerais	13.18	38.03	305.81	398.10	22.60	375.50
Rio Grande do Sul	17.54	52.77	421.37	503.47	31.30	472.17
Distrito Federal	14.79	49.83	403.02	393.29	29.97	363.32

Table 2 we can observe the income inequality, per capita income and the IDHM of the federative units according to socioeconomic levels. It is possible to observe that among all the studied units, the highest value of income inequality was found in a federative unit classified as low socioeconomic level (Bahia, Gini = 0.60) although in this federative unit there is the highest per capita income among the federative units classified as low socioeconomic status. Among the UFs classified as moderate socioeconomic level, Amazonas stands out for presenting lower HDI, lower per capita income and for being among the federative units with the second highest value of the Gini index. This behavior observed in Amazonas is repeated for the Federal District among the federative units classified as high socioeconomic level (Table 2).

**Table 2:** Description of the socioeconomic variables of the study on mortality from female breast cancer in the federal units of Brazil, 2017.

**Socioeconomic** **levels/Federative units**	**HDI**	**Gini**	**per capita income** **(R$)**
**Low**			
Bahia	0.71	0.60	566.60
Maranhão	0.68	0.54	387.70
Pará	0.69	0.53	468.48
Sergipe	0.70	0.56	541.98
Alagoas	0.68	0.53	426.33
Acre	0.71	0.57	498.02
Piauí	0.69	0.54	487.40
**Moderate**			
Amapá	0.74	0.59	605.04
Roraima	0.75	0.55	650.51
Ceará	0.73	0.56	538.22
Mato Grosso do Sul	0.76	0.48	841.32
Pernambuco	0.72	0.56	558.98
Espírito Santo	0.77	0.51	800.14
Mato Grosso	0.77	0.47	809.58
Tocantins	0.74	0.50	610.38
Rondônia	0.72	0.46	619.23
Rio Grande do Norte	0.73	0.53	550.17
Amazonas	0.73	0.60	558.03
Goiás	0.76	0.49	835.77
Paraíba	0.72	0.56	601.71
**High**			
Rio de Janeiro	0.79	0.52	960.11
Santa Catarina	0.80	0.42	1044.59
Paraná	0.79	0.49	968.39
São Paulo	0.82	0.53	1134.12
Minas Gerais	0.78	0.50	804.61
Rio Grande do Sul	0.78	0.49	1073.13
Distrito Federal	0.85	0.59	1688.49

When we adjust the relationship between the socioeconomic indicators and the epidemiological indicators of breast cancer by the HDI and per capita income (model 1), only the federative units with low socioeconomic status showed statistically significant results (*p* <0.05) for all indicators [mortality, β = −13.7 (95% CI −24.4; −3.0); *r*^2^ = 0.84 *p* = 0.023; incidence, β = −39.1 (−63.5; −14.7), *r*^2^ = 0.91; *p* = 0.011; prevalence, β = 301.1 (−469.9; −132.2), *r*^2^ = 0.92; *p* = 0.008; DALY β = −463.5 (−750.4; −176.7), *r*^2^ = 0.90; *p* = 0.001; YLD, β = −22.9 (−36.5; −9.3), *r*^2^ = 0.92; *p* = 0.009 and; YLL, β = −440.6 (−714.2; −167.0) *r*^2^ = 0.89; *p* = 0.001] (Table 3).

**Table 3:** Impact of income inequality on breast cancer mortality according to socioeconomic status in the federative units of Brazil in 2017.

**Socialeconomic levels/** **Characteristics**	**Model 1** ^*^				**Model 2** ^**^			
	**β (IC 95%)**	**r^2^**	p^*^	**p^+^**	**β (IC 95%)**	**r^2^**	p^**^	**p^+^**
**Low**								
Mortality	−13.7(−24.4; −3.0)	0.84	0.023	0.658	−15.4(−31.0; 0.3)	0.86	0.053	0.320
Incidence	−39.1(−63.5; −14.7)	0.91	0.011	0.707	−44.0(−78.9; −9.9)	0.92	0.026	0.320
Prevalence	−301.1(−469.9; −132.2)	0.92	0.008	0.749	−341.0(−565.9; −116.2)	0.95	0.017	0.320
DALYs	−463.5(−750.4; −176.7)	0.90	0.011	0.676	−546.1(−892.3; −199.9)	0.94	0.015	0.320
YLDs	−22.9(−36.5; −9.3)	0.92	0.009	0.725	−26.0(−44.5; −7.5)	0.93	0.021	0.320
YLLs	−440.6(−714.2; −167.0)	0.89	0.011	0.671	−520.1(−848.0; −192.2)	0.93	0.015	0.320
**Moderate**								
Mortality	−3.2(−17.4; 11.1)	0.03	0.630	0.336	– 4.3(−20.1; 11.5)	0.06	0.552	0.590
Incidence	−9.0(−46.1; 28.1)	0.04	0.601	0.309	−10.4(−52.0; 31.1)	0.05	0.584	0.659
Prevalence	−69.5(−358.6; 219.6)	0.05	0.604	0.357	−77.9(−402.5; 246.7)	0.06	0.600	0.672
DALYs	−117.4(−559.1; 324.3)	0.04	0.567	0.406	−146.2(−638.1; 345.6)	0.06	0.518	0.576
YLDs	−5.3(−26.9; 16.3)	0.05	0.594	0.328	−6.1(−30.3; 18.2)	0.06	0.586	0.657
YLLs	−112.1(−532.7; 308.6)	0.04	0.566	0.414	−140.2(−608.3; 327.9)	0.06	0.515	0.573
**High**								
Mortality	−5.2(−32.6; 22.2)	0.07	0.625	0.702	−5.4(−41.4; 30.5)	0.09	0.663	0.320
Incidence	−10.0(−85.3; 65.2)	0.13	0.730	0.600	−102.1(−109.9; 89.5)	0.14	0.766	0.320
Prevalence	−67.4(−635.9; 501.00)	0.17	0.758	0.555	−67.9(−822.3; 686.5)	0.17	0.793	0.320
DALYs	−117.9(−908.7; 672.7)	0.15	0.700	0.694	−123.3(−1164.8; 918.1)	0.16	0.731	0.320
YLDs	−5.1(−49.5; 39.3)	0.15	0.766	0.597	−5.2(−64.1; 53.7)	0.15	0.798	0.320
YLLs	−112.9(−859.8; 634.0)	0.17	0.696	0.699	−118.1(−1101.3; 865.0)	0.19	0.728	0.320

* Linear Regression by HDI and capita income;

** Model 1 adjusted by the Gini index; ^+^ White's test.

With income inequality included in the model, most epidemiological indicators remain reduced and significant, except for mortality. Adjusting the model for income inequality, the reduction in mortality observed in model 1 is no longer significant [β = −15.4 (95% CI −31.0; 0.3); *r*^2^ = 0.86; *p* = 0.053], demonstrating the income inequality impact on the effects of increased socioeconomic development and per capita income on breast cancer mortality (Table 3).

## Discussion

When analyzing the relationship between income inequality and epidemiological indicators of breast cancer mortality in women according to socioeconomic levels of Brazilian federative units with low socioeconomic levels, it was observed that there is an impact of income inequality on the effects of increased socioeconomic development and per capita income on breast cancer mortality. The HDI is important for improving the health of populations, as it reflects quality health, access to health services, as well as other positive indicators. The lack of health resources is worrisome, since it demonstrates that in most UFs there are no resources to finance an adequate health infrastructure to provide assistance and a lack of qualified health professionals (16, 17).

The Federative Units in Brazil present intraregional gaps that become more evident by social, economic, geographical, and cultural issues. The scarce access to certain distant locations and the lack of technology in the health care system impact the indicators of lifestyle for the more vulnerable population, making the diagnosis of breast cancer challenging (7).

Several epidemiological studies have identified individual, lifestyle and environmental conditions that increase the likelihood of developing breast cancer, as risk factors: heredity, reproductive, age and race that cannot be changed (18–20).

Other environmental or behavioral factors, such as hormone replacement, alcohol intake, excess body fat or tobacco use can be reduced (21, 22). Regular physical activity and breastfeeding are also ways to protect yourself from breast cancer (23).

There is a plan of priority actions foreseen in the Strategic Action Plan for Coping NCDs in Brazil, from 2011 to 2022 which are: screening, expanding access to mammography for women aged 50 to 69 years; mammography quality; Early Diagnosis; Timely and Quality Treatment; Communication and Social Mobilization; Professional Training and Epidemiological Information (24).

The clinical stage of the diagnosis is an important predictor that contributes to the success of the breast cancer treatment. In Brazil, this factor is influenced by the type of health care (public or private). In the public health care system, only 15% of patients are diagnosed during the initial stages of the disease, while 33% of the patients find themselves in this condition in the private sector, which directly affects the treatment outcome (25).

The time between the diagnosis and the beginning of the treatment is a factor that distinguishes both groups, since this interval tends to be shorter in the private sector. This gap between public and private health care systems might constitute a factor that influences the mortality rates in both groups, as well as occurrence and prevalence of breast cancer. Whereas, the South and Southeast regions of Brazil reach the highest health care insurance coverage with over 30% of the population, the North and Northeast regions present < 13% coverage rate, which can impose an obstacle in the tracking of the disease and an increase in its severity (26, 27).

Rosa et al. (28) have shown that, in the public health care system, the method for detecting breast cancer is usually the evaluation of symptoms, while in the private sector women are diagnosed *via* check-up exams. That shows how urgent it is to create public policies that aim at spreading the access to mammographic tracking to women from all the Federative Units in the country, as a strategy to decrease the gap among breast cancer mortality rates throughout the most vulnerable regions of Brazil (28).

Income inequality can impact breast cancer indicators such as mortality due to risk factors for breast cancer and the lack of access to the service due to lack of knowledge. Income inequality is likely to increase because Brazil in 2010 showed low trends in income inequality. Between 2001 and 2011, average household income grew by more than 30%, inequality as measured by the Gini coefficient fell by more than 10% (29).

However, in the next decade, the country experienced a loss of control over public accounts, the worst recession since the redemocratization. The year 2015 was the watershed. This instability affected different forms of income. Between 2016 and 2018 the highest level of inequality in the series occurred: 0.545. In 2020 the recent crisis triggered by the COVID-19 pandemic (30).

The limitations of the present study are related to the collection of secondary data, which do not represent individuals and bring possible biases. It is suggested that this study be conducted with other designs, such as cohort, for example, to evaluate the effect of these relationships found at another level of analysis.

## Conclusion

In the present study, it was concluded that there is an impact of income inequality on the effects of increased socioeconomic development and per capita income on breast cancer mortality, where income inequality is prone to poor socioeconomic conditions, and thus having worse conditions of having functional health, characterizing the highest mortality from breast cancer in the Brazilian federative units in the year 2017.

It was found that the income inequality might explain the association between income and breast cancer mortality in the lower HDI tertiles, there is need for adequate public policies for breast cancer that can have preventive exams from the age of 40, more information and access to treatments, put into practice the action plans for the population of low socioeconomic status.

## Data availability statement

Publicly available datasets were analyzed in this study. This data can be found at: http://ghdx.healthdata.org/gbd-results-tool.

## Author contributions

KT conceived the study, analyzed the data, constructed the results from the data, and wrote the manuscript. SF analyzed the data, constructed the results from the data, and wrote the manuscript. WM collected the data and constructed the results from the data. FF conceived the study, analyzed the data, and reviewed results. FA conceived the study and reviewed the results. All authors read and approved the final manuscript.

## Funding

This work was supported by National Council for Scientific and Technological Development (CNPq)-FA was CNPq research productivity scholarship (Process Number 307183/2018-1).

## Conflict of interest

The authors declare that the research was conducted in the absence of any commercial or financial relationships that could be construed as a potential conflict of interest.

## Publisher's note

All claims expressed in this article are solely those of the authors and do not necessarily represent those of their affiliated organizations, or those of the publisher, the editors and the reviewers. Any product that may be evaluated in this article, or claim that may be made by its manufacturer, is not guaranteed or endorsed by the publisher.
